# A Phase IIb, randomized, multicenter study of the efficacy of GVAX pancreas vaccine and CRS-207 compared to chemotherapy or to CRS-207 alone in adults with previously-treated metastatic pancreatic adenocarcinoma (eclipse study)

**DOI:** 10.1186/2051-1426-2-S3-P68

**Published:** 2014-11-06

**Authors:** Andrea Wang-Gillam, Vincent Picozzi, Todd Crocenzi, Michael Morse, Herbert Zeh, Robert Fine, Aimee Murphy, Justin Skoble, Edward Lemmens, Sandy Ferber, Allan Rosen, John Grous, Thomas W Dubensky, Dirk Brockstedt, Elizabeth Jaffee, Dung Le

**Affiliations:** 1 Washington University School of Medicine in St. Louis, St. Louis, MO, USA; 2 Virginia Mason Medical Center, Seattle, WA, USA; 3 Earle A. Chiles Research Institute, Portland, OR, USA; 4 Duke University Medical Center, Durham, NC, USA; 5 University of Pittsburgh Medical Center, Pittsburgh, PA, USA; 6 Columbia University, New York, NY, USA; 7 Aduro BioTech, Inc., Berkeley, CA, USA; 8 The Sidney Kimmel Comprehensive Cancer Center at Johns Hopkins, Baltimore, MD, USA

## Background

A heterologous prime-boost vaccination strategy using GVAX pancreas vaccine and CRS-207 is showing promise in patients with metastatic pancreatic adenocarcinoma (PDA). GVAX is composed of lethally-irradiated, allogeneic pancreatic cancer cells modified to express GM-CSF and induces a broad response against multiple tumor antigens. GVAX is given with low-dose cyclophosphamide (CY) to inhibit regulatory T cells. CRS-207 is live-attenuated *Listeria monocytogenes *engineered to express the tumor-associated antigen mesothelin. CRS-207 boosts responses against mesothelin and is unique in its capacity to stimulate both innate and adaptive immunity by activating T cells and NK cells. In a recently completed Phase II study, the CY/GVAX plus CRS-207 combination resulted in statistically-significant improvement of overall survival (OS) compared to CY/GVAX alone (Le, GI ASCO 2014).

## Methods

This is a Phase IIb study comparing CY/GVAX and CRS-207 to chemotherapy or to CRS-207 alone in adults with previously-treated metastatic PDA. Subjects will be enrolled in two cohorts: 150 subjects into a primary cohort of patients with at least two prior treatment regimens for metastatic disease (3^rd^+ line) and 90 subjects into an exploratory cohort of patients with only one prior treatment regimen for metastatic disease (2^nd ^line). Subjects will be randomized in a 1:1:1 ratio to receive either 2 doses of CY/GVAX and 4 doses of CRS-207 (Arm A), 6 doses of CRS-207 (Arm B) or physician's choice of select single-agent chemotherapy (Arm C). The primary objective is to compare OS in the primary cohort between Arms A and C. Secondary/exploratory objectives include: comparison of OS in both primary and exploratory cohorts between all treatment arms, assessment of safety and clinical responses (tumor assessments and CA19-9 levels) and correlation of *Lm- *and mesothelin-specific T cell and other immunological responses with OS, progression-free survival and best overall response. (Sponsor: Aduro BioTech, Inc.; NCT02004262).

